# SGLT-2 inhibitors and atrial fibrillation in the Food and Drug Administration adverse event reporting system

**DOI:** 10.1186/s12933-021-01243-4

**Published:** 2021-02-11

**Authors:** Benedetta Maria Bonora, Emanuel Raschi, Angelo Avogaro, Gian Paolo Fadini

**Affiliations:** 1 Department of Medicine, University of Padova, Via Giustiniani 2, 35128 Padova, Italy; 2 Department of Medical and Surgical Sciences, University of Bologna, Bologna, Italy

**Keywords:** Pharmacovigilance, Epidemiology, Complications, Observational, Type 2 diabetes, Clinical practice

## Abstract

**Background:**

Sodium glucose cotransporter-2 inhibitors (SGLT2i) reduce the risk of heart failure and new data show they can prevent atrial fibrillation (AF). We examined the association between SGLT2i and AF in the Food and Drug Administration adverse event reporting system (FAERS).

**Methods:**

We mined the FAERS from 2014q1 to 2019q4 to compare AF reporting for SGLT-2 i versus reports for other glucose lowering medications (ATC10 class). Several exclusions were sequentially applied for: concomitant medications; diabetes, cardiovascular or renal disease indication; reports for competing adverse events (genitourinary tract infections, ketoacidosis, Fournier’s gangrene, amputation). We provide descriptive statistics and calculated proportional reporting ratios (PRR).

**Results:**

There were 62,098 adverse event reports for SGLT2i and 642,031 reports for other ATC10 drugs. The reporting of AF was significantly lower with SGLT2i than with other ATC10 drugs (4.8 versus 8.7/1000; p < 0.001) with a PRR of 0.55 (0.49–0.62). Results did not change substantially after excluding reports listing insulin (PRR 0.49) or anti-arrhythmics (PRR 0.59) as suspect or concomitant drugs, excluding reports with indications for cardiovascular disease (PRR 0.49) or renal disease (PRR 0.55), and those filed for competing adverse events (PRR 0.63). Results were always statistically significant whether the diabetes indication was specified. Negative and positive controls confirmed internal validity of the database.

**Conclusions:**

In a large pharmacovigilance database, AF was robustly and consistently reported more frequently for diabetes medications other than SGLT2i. This finding complements available evidence from trials supporting a protective role of SGLT2i against the occurrence of AF.

## Background

Inhibitors of sodium glucose cotransporter-2 (SGLT2i) lower the renal threshold for glucose resorption in the proximal renal tubule, thereby causing glycosuria. In patients with type 2 diabetes (T2D), SGLT2i are effective in controlling glycemia, blood pressure, and body weight [1]. According to the results of large cardiovascular outcome trials (CVOTs), SGLT2i prevent hospitalization for heart failure (HHF) in patients with T2D with or without a prior history of HF or cardiovascular disease (CVD) at baseline [2]. In two trials performed on patients with HF and reduced ejection fraction (HFrEF), 42–50% of whom had T2D, SGLT2i significantly improved HF outcomes [3, 4].

Interestingly, atrial fibrillation (AF) has been shown to occur less frequently among patients who received dapagliflozin than among those who received placebo in the DECLARE trial (HR 0.81; 95% CI 0.68–0.95) [5]. Other studies have reported similar lower rates of AF among patients randomized to SGLT2i, and two meta-analysis calculated a 21% relative risk reduction [6, 7]. This finding is relevant because T2D is an established risk factor for AF [8, 9], which can cause embolic stroke, precipitate HF [10], or result in hospitalization for the need of rate control. Therefore, though mechanisms of this association are unclear [11], preventing AF in people with diabetes can exert positive effects on global outcomes.

To date, whether such benefit observed in trials applies to clinical practice is unknown. The real-world setting differs from the experimental trial setting in many instances, including the way outcomes, like AF, are screened, ascertained, adjudicated, and reported. Thus, it is important that, in the absence of dedicated trials, potential benefits resulting from post-hoc trial analyses are confirmed in clinical practice. So far, limited real-world studies provided inconsistent results on the association between use of SGLT2i and rates of AF [12–14]. Spontaneous reports of adverse events (AE) populate large databases with clinically-relevant information emerging from clinical practice. The analysis of pharmacovigilance databases can inform on the associations between drugs and health outcomes defined by AE. Though pharmacovigilance is traditionally used to detect signals of potential harm, more recently, spontaneous reporting systems have been exploited to uncover patterns indicative of reduced reporting (positive effects), e.g. for drug repurposing [15, 16]. Furthermore, the consistency between trial results and pharmacovigilance analyses has been recently confirmed, thus raising the debate on the role of spontaneous reports as potential source of risk estimates under certain circumstances [17]. Therefore, AE reporting databases can be re-used as a source of real-world evidence that complement available information from trials and traditional cohort studies. This approach has been successfully used to explore outcomes associated with SGLT2i and other diabetes therapies, not limited to the detection of rare AE [18–21].

Herein, we examined a large pharmacovigilance database to evaluate the reporting frequency of SGLT2i with AF as compared to other glucose-lowering medications.

## Methods

### Data source

The Food and Drug Administration (FDA) adverse event reporting system (FAERS) receives reports of drug-related AE from healthcare professionals, drug manufacturers, and consumers from all over the world. The FDA curates and maintains the database and makes FAERS files publicly available on a quarterly basis. Each quarterly archive contains orthogonal files with data on patient characteristics, description and type of AE, suspect or concomitant drugs and their indications for use, AE outcomes, and reporting source. Each report is composed by data on patient demographics (age, sex, country), one or more AE (reactions attributed to one or more drugs), one or more drug(s) considered to be suspect or concomitant, the indication(s) for use of such drugs, severity and outcomes of the reaction(s). Extensive elaboration and mining of these files is needed to map all relevant data to the correct AE report, identify and exclude duplicates, and make AE reports searchable. We used AERSmine, a validated web-based platform that searches AE reports within FAERS files based on filters for drugs, reactions, indications and other characteristics including reporting period, source, and demographics. AERSmine operates a systematic normalization, unification, and ontological aggregation of the drugs, indications, and AE, thus allowing comparisons of large cohorts based on exposure [22].

### Exposure and outcome

We set the exposure time between 2014q1 and 2019q4 because the number of AE reports filed for SGLT2i was negligible before 2014 and AERSmine was updated to the last quarterly FAERS archive of 2019. In each analysis, we selected two groups of AE reports: one listing SGLT2i as suspect or concomitant (active treatment group) and the other listing any other diabetes medication as suspect or concomitant (control treatment group). The group of SGLT2i was composed as follows: “canagliflozin” OR “empagliflozin” OR “dapagliflozin” OR “metformin and canagliflozin” OR “linagliptin and empagliflozin” OR “metformin and empagliflozin” OR “ertugliflozin” OR “ipragliflozin” OR “metformin and dapagliflozin” OR “sotagliflozin”. Diabetes medications map to the anatomic therapeutic classification (ATC) A10 class and contain insulin (A10A) and non-insulin drugs (A10B). Details on the terms used for diabetes medications can be found in the Appendix. The AE of interest (AF) was composed by the terms “atrial fibrillation” and “atrial flutter”, because the two entities can sometimes be hardly distinguished clinically. We retrieved the total number of reports in the two groups and the number of reports containing AF as an AE. We extracted information on demographics (age category and sex), whether the SGLT2i or control drug was suspect or concomitant in the AE report, and type of the reporting source.

### Sensitivity analyses

We used a series of exclusions to evaluate robustness of the findings and whether typical confounders of pharmacovigilance analysis may have biased results [23]. First, because drugs indicated for the treatment of diabetes may sometimes be used by non-diabetic individuals (e.g. metformin for polycystic ovary syndrome or GLP-1 receptor agonists for obesity), all analyses were performed in duplicate with or without filtering for the diabetes indication. The main analysis was repeated excluding from both groups reports listing anti-arrhythmics as suspect or concomitant drugs to avoid reverse causality in the association with AF, or excluding those listing renal disease (acute or chronic) as indications, because AF may occur preferentially in patients with renal disease [24], for whom SGLT2i have long been contraindicated. Further, since use of insulin is typically considered a proxy of disease severity or advanced disease stage, we excluded AE reports for insulin from the control group. Downstream of this filter, we excluded AE reports with an indication for CVD, because AF might occur preferentially in patients with known CVD, who are more likely to receive a SGLT2i according to guidelines [25]. Finally, we excluded competing AE that are typically reported preferentially among users of SGLT2i [genitourinary tract infections (GUTI), diabetic ketoacidosis (DKA), amputations, and Fournier’s gangrene (FG)], in order to avoid the bias due to dilution/competition [26]. Additionally, we checked consistency of FAERS results using internal controls. As positive control of AF signal, we examined its association with ibrutinib, as reported in the literature [27]. As negative controls for SGLT2i, we verified no signal for a falsification AE not expected to be associated with SGLT2i (appendicitis) and no signal for a typical cardiovascular outcome not affected by SGLT2i in trials (stroke) [2]. As positive controls for SGLT2i, we elected heart failure and chronic kidney disease [2]. Details on search terms, needed to replicate the analyses, are given in  Additional file 1.

### Statistical analysis

Descriptive data are reported as percentage of patients within each category of age, sex, drug (suspect or concomitant) and reporting source. Numbers of reports in each group were used to calculate the proportional reporting ratio (PRR) with 95% confidence interval (CI) as previously described [28]. Comparison of rates was performed using the chi square test. Statistical significance was accepted at p < 0.05.

## Results

### Numbers and characteristics of reports

**Fig. 1:**
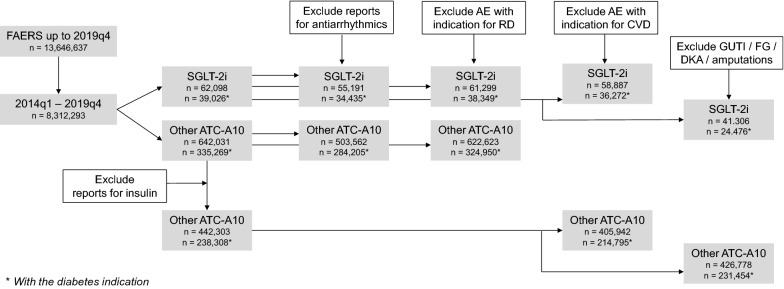
Study flow chart. A series of interconnected analyses is reported, with progressive exclusions. FAERS, FDA adverse event reporting system. SGLT2i, sodium glucose cotransporter-2 inhibitors. *ATC* anatomical therapeutic classification, *AE* adverse event, *CVD* cardiovascular disease, *RD* renal disease, *GUTI* genito-urinary tract infections, *DKA* diabetic ketoacidosis, *FG* Fournier’s gangrene. Numbers are referred to the total number or reports in each analysis (*with the diabetes indication)

**Fig. 2:**
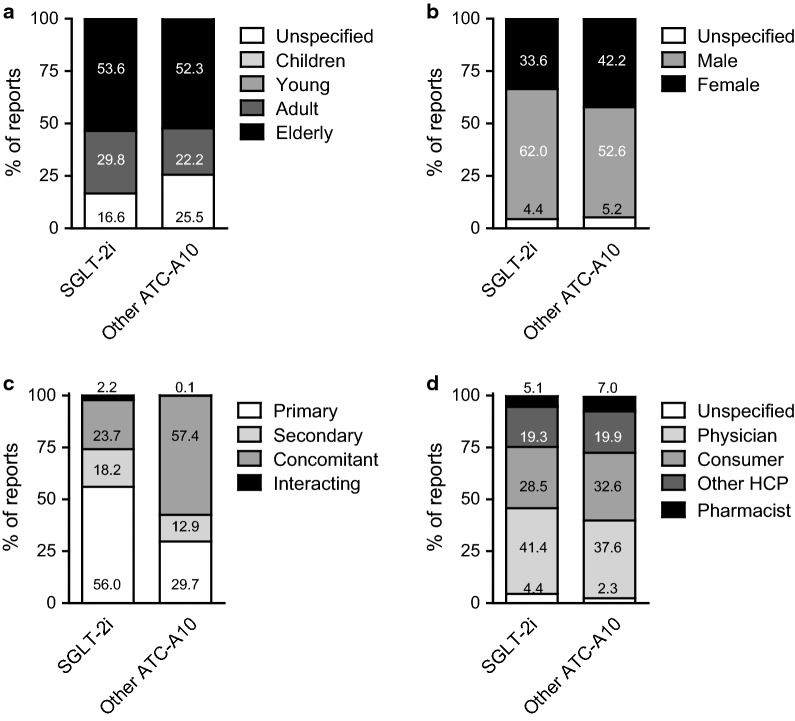
Case demographics. Key characteristics of the AF reports are shown: age category (**a**), sex (**b**), drug role (**c**), reporting source (**d**). SGLT2i, sodium glucose cotransporter-2 inhibitors. *ATC* anatomic therapeutic classification, *HCP* health care professional. Numbers inscribed within stacked bars are percentages

The FAERS up to 2019q4 is populated by 13,646,637 spontaneous AE reports. In the period when SGLT2i were available (2014q1–2019q4), there were 8,312,293 total reports, 62,098 of which contained at least one SGLT2i as suspect or concomitant and 642,031 contained at least another diabetes drug of the ATC class A10, including insulin (Fig. 1). Figure 2 shows patient demographics described in AF reports listing SGLT2i or other ATC-A10 class drugs as suspect or concomitant. More than 50% of patients were aged 65 years or older in both groups of reports, but patients on SGLT2i were more often males than those on other ATC-A10 class drugs. SGLT2i were considered primary suspect of the reported AE more often than other ATC-A10 class drugs (56.0% vs. 29.7%), which were more often listed as concomitant medications. For both groups of reports, the most common source were physicians or other health care professionals, while about 30% of reports were filed by patients (Fig. 2).

### Disproportionality analysis

**Fig. 3:**
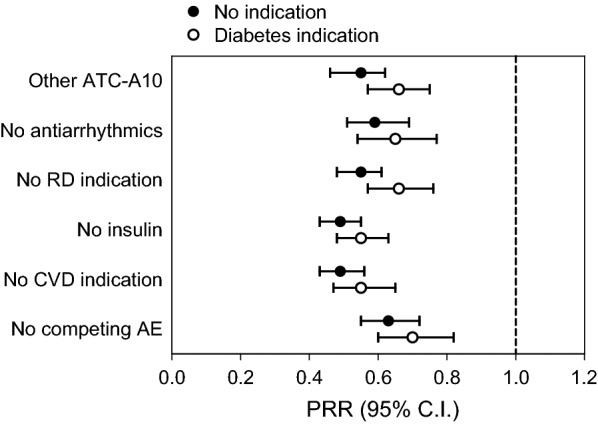
Disproportionality analysis. The Forest plot shows proportional reporting ratios (PRR) with 95% confidence intervals (CI) for atrial fibrillation (AF) in reports for sodium glucose cotransporter-2 inhibitors (SGLT2i) versus control drugs. A PRR < 1.0 indicates a disproportional lower rate of AF among reports for SGLT2i. *ATC* anatomic therapeutic classification, *AE* adverse event, *CVD* cardiovascular disease, *RD* renal disease

Among reports for SGLT2i, 295 contained AF as an AE (proportion 4.8/1000), whereas among reports for other ATC-A10 drugs, 5565 contained AF as an AE (8.7/1000). The corresponding PRR was 0.55 (95% CI 0.49–0.62; p < 0.001; Fig. 3), implying that AF was reported disproportionally less frequently in association with SGLT2i than with other diabetes drugs. Such disproportionality was highly consistent among individual molecules: 0.47 (95% CI 0.39; 0.56) for canagliflozin; 0.51 (95% CI 0.40–0.65) for dapagliflozin; 0.71 (95% CI 0.59; 0.86) for empagliflozin.

Restricting the search to reports where the two groups of drugs were identified as primary suspect still yielded lower reporting of AF associated with SGLT2i (3.8/1000) versus other ATC-A10 class (5.7/1000), with a PRR of 0.66 (95% CI 0.57–0.77). A lower reporting of AF for SGLT2i than among those for other ATC-A10 class drug was also detected after restricting to reports filed by physicians or other healthcare professionals, with a PRR of 0.43 (95% CI 0.37–0.50). There was no difference in the PRR of AF associated with SGLT2i versus other ATC-A10 class drugs between males (0.56; 95% CI 0.48–0.65) and females (0.47; 95% CI 0.38–0.57).

### Sensitivity analyses

We refined the search in a series of additional sensitivity analyses to evaluate robustness of the finding (Fig. 1). To avoid reverse causality (e.g. AF showing up less frequently among reports for SGLT2i being restricted to reports for other drugs), we excluded from both groups reports listing anti-arrhythmics as suspect or concomitant drugs. PRR remained largely and significantly in favour of SGLT2i (0.59; 95% CI 0.51; 0.69) even when reports for patients taking anti-arrhythmic drugs were excluded. Further, since kidney disease is a risk factor for AF [24] and was long considered a contraindication to SGLT2i use, we excluded all reports wherein acute or chronic renal disease was an indication. Even with this exclusion, disproportionality in favour of SGLT2i did not change substantially (PRR 0.55; 95% CI 0.48; 0.61). In the analysis excluding insulin as a proxy of disease severity, the total number of reports for the control group was reduced by 31 % (to 442,303) but the reporting of AF remained significantly lower for SGLT2i (4.8/1000 vs. 9.7/1000; p < 0.001) and the PRR was 0.49 (95% CI 0.43–0.55). Furthermore, to evaluate whether the lower rates of AF associated with SGLT2i was linked to the patient’s history of CVD, we excluded all reports listing at list one drug with the indication for CVD. In this search enriched of AE reports for patients without CVD, the rate of AF associated with SGLT2i was still lower than that associated with other non-insulin ATC-A10 drugs (PRR 0.49; 95% CI 0.43–0.56). Finally, to rule out that AF appeared less frequently in reports for SGLT2i because of dilution by other AEs, we excluded reports for GUTI, DKA, FG and amputations from the analysis. The PRR still indicated less AF reports among users of SGLT2i versus other diabetes drugs (0.61; 95% CI 0.55–0.72). For all these analyses, restricting the search to reports wherein the diabetes indication was specified for SGLT2i and control ATC-A10 drugs yielded always significantly lower AF reporting for SGLT2i with PRR < 1.0 (Fig. 3). When all the filters and exclusions illustrated in Fig. 1 were applied at the same time, the number of reports decreased substantially, but the PRR still indicated a lower AF reporting associated with SGLT2i compared with control diabetes drugs (PRR 0.75; 95% CI 0.59; 0.95).

Internal controls were assessed to check consistency within the database. The PRR for appendicitis, not expected to be affected by SGLT2i, was 1.18 (95% CI 0.78–1.79). Reports for stroke showed no disproportional association with SGLT2i versus other ATC10 class drugs (PRR 0.93; 95% CI 0.79; 1.09), while there was a significant reduced reporting of heart failure (PRR 0.16; 95% CI 0.15; 0.18) or chronic kidney disease (PRR 0.32; 95% CI 0.28; 0.36) for SGLT2i. Finally, there was a clear disproportionality of AF reporting associated with use of ibrutinib (PRR 10.6; 95% CI 10.2–11.1), confirming literature data [27].

## Discussion

We show that, in one of the world largest pharmacovigilance databases, AF was reported disproportionally less frequently among patients using SGLT2i than among patients using other glucose-lowering medications. This result was highly consistent in several sensitivity analyses performed to reduce the possibility of bias, including the use of negative and positive controls.

Along with the results of individual randomized controlled trials (RCTs) and meta-analyses [6, 7], this finding supports the suggested protective effect of SGLT2i against AF. To date, observational studies have provided conflicting results. The CVD-Real Nordic, a retrospective observational study (n = 40,908 patients) performed in Denmark, Norway and Sweden, reported no difference in the incidence of AF among patients who received SGLT2i versus matched patients who received DPP-4 inhibitors [13] or other glucose-lowering medications [12]. On the other side, a study from Taiwan reported markedly lower rates of AF among 15,606 new users of SGLT2i versus 12,383 new users of DPP-4 inhibitors, after inverse probability of treatment weighting [14]. In view of these conflicting results, more real-world data are needed to verify whether the protection exerted by SGLT2i against AF observed in trials could apply to clinical practice. Our new analysis adds further evidence because spontaneous AE reporting databases can be used to explore health outcome in the real-world. We contribute to extend the cumulative knowledge about the safety and effectiveness of SGLT2i in an unselected population using a global pharmacovigilance database, thus supporting generalizability of trial findings.

Though generally considered a relatively benign arrhythmia, new-onset AF is burdened by significant morbidity and mortality, driving markedly elevated risk for stroke, dementia, HF, and overall mortality [29–31]. Due to its high healthcare and societal costs, preventing AF has become a major public health priority [32]. Thus, prevention of AF by SGLT2i can yield substantial benefits on patients’ outcomes and quality of life. One could even argue that the effect against AF drives part of the extraordinary cardiorenal benefits observed during therapy with SGLT2i in patients with and without diabetes [2–4, 33].

The mechanisms driving occurrence of AF are still debated, but include atrial fibrosis and electrical remodelling, with or without the coexistence of triggering ectopic foci [34]. There are several potential mechanisms whereby SGLT2i may reduce the incidence of AF [35]. SGLT2i can prevent glucotoxicity in the heart, switching cardiac metabolism to metabolically favourable substrates. Reduction of pre-load, decongestion and reduction of filling pressures, achieved by the peculiar diuretic effect of SGLT2i, would blunt the stress imposed to atrial cardiomyocytes. Also, thinning of epicardial adipose tissue, resulting from loss of body weight and fat mass, may spare atria from pro-inflammatory signals driving remodelling and fibrosis [36]. Finally, SGLT2i can exert direct electrophysiological effects by modulating sodium handling and mitochondrial function [37, 38], which may counteract AF triggers.

We wish to underline some important characteristics of pharmacovigilance studies and their intrinsic limitations. First, these studies differ from observational cohort research mainly because the population is composed of patients for whom at least one AE was filed for at least one drug, thereby missing information on the background population of individuals exposed to the drug(s) but not reporting any AE. If no true association exists between a given drug and an AE, such AE should have the same frequency in reports listing such drug as in other reports, yielding no disproportionality. Indeed, pharmacovigilance data cannot be used to derive absolute incidence of an event, but only to compare reporting of an event in association with different drugs. Another important limitation is that clinical-level data (such as HbA1c, BMI, kidney function) are not available in pharmacovigilance databases, preventing full assessment of comparability of patient characteristics within the various AE groups. For these reasons, after having shown a disproportionally lower rate of AF among reports for SGLT2i, we undertook a series of sensitivity analyses and internal controls to verify robustness of the finding. A positive control for AF (ibrutinib), as well as negative and positive control AE for SGLT2i confirmed internal validity of the method. Excluding reports for anti-arrhythmic drugs re-assured on competition with SGLT2i for AF, whereas excluding AE reporting renal disease as a drug indication reasonably ruled out that AF occurred less frequently in reports for SGLT2i because chronic kidney disease, a risk factor for AF, was long considered a contraindication to SGLT2i. Exclusion of reports for insulin allowed to rule out that the control group of drugs were being prescribed to sicker patients, driving a spurious association. Excluding patients with an indication for CVD ruled out that the finding was driven by patients who were already affected by cardiac problems, who are more likely to receive SGLT2i. Considering that only part of AE reports filed for glucose-lowering medications specify the diabetes indication and because some diabetes drugs could be used by non-diabetic individuals, all analyses were repeated excluding reports without the diabetes indication. In all cases, AF was reported disproportionally less frequently in association with SGLT2i than with control drugs. Pharmacovigilance traditionally aims to discover signals of potentially new drug-associated AEs, while its ability to reliably inform on inverse associations indicative of risk reduction is still debated. In the case of SGLT2i, their consolidated use in clinical practice and largely characterized safety profile make spontaneous reporting a likely indicator of risk in clinical practice, provided that major reporting biases are reasonably excluded with available methods. Dilution by competing AEs is of concern. In other term, the rate of AF among reports for SGLT2i might be diluted by common and well-recognized SGLT2i-associated AE, increasing denominator of the PRR. To address this issue, we repeated the analysis by excluding common AE associated with SGLT2i and AE that are less common but highly specific for SGLT2i. Even with such exclusion, on top of insulin exclusion and for both reports with and without the diabetes indication, disproportionality remained robust and significant. Remarkably, even after combining all the above-mentioned filters and exclusions together, AF rates remained 25% significantly lower among reports for SGLT2i than for other diabetes drugs.

Among other limitations, we wish to acknowledge that duration of treatment (time elapsed from drug initiation to AE occurrence) is described in a minority of reports in the FAERS. In addition, there is no mean of assessing how AF was diagnosed, e.g. based on symptoms or instrumental screening.

In face of these limitations, to strengthen the possible causal connection between use of SGLT2i and the lower rates of AF, we globally assess the evidence using Bradford Hill criteria (Table 1): with the exception of some items that we could not assess (namely biological gradient and reversibility), all criteria appear to be satisfied thereby supporting, though not proving, a cause-effect relationship.

**Table 1:** Bradford Hill criteria for causation

Criterion	Score	Note
Strength	Moderate	The rate of AF among reports for SGLT2i was about halved compared to those for control drugs, or is about 2× for control drugs versus SGLT2i
Consistency	High	Disproportionality changed little and remained significant in all sensitivity analyses. Therefore, the impact of unmeasurable confounders is likely to be negligible
Specificity	Good	Positive and negative controls were satisfied
Temporality and reversibility	Not assessed	Time from drug initiation to AE occurrence, as well as information on drug withdrawal and re-challenge was not available
Biological gradient	Not assessed	We had no data on the dosage of SGLT2i. However, the gradient of SGLT2i dose is limited (max 2.5-fold)
Plausibility	Satisfied	There are several potential mechanisms whereby SGLT2i may reduce the risk of AF
Coherence	Good	Lower rates of AF among patients taking SGLT2i have been reported in randomized control trials, and in one of two observational studies
Experimental support	Satisfied	Randomized controlled trials provide the most compelling experimental evidence for protection against AF by SGLT2i. Yet, it should be noted that none of such trials had AF as primary endpoint
Analogy	Good	The FAERS has been used previously to demonstrate associations between AF rates and other specific drugs (e.g. ibrutinib)

## Conclusions

In summary, our analysis of a large pharmacovigilance database indicates a consistent and robust reduced reporting of AF with SGLT2i, thus adding further evidence towards a real protective affect against AF, as observed in trials. Considering all the evidence available and the limitations intrinsic to our approach, dedicated prospective observational real-world studies are needed to confirm definite transferability to clinical practice. In addition, it is worth investigating whether SGLT2i can protect against AF also in patients without diabetes.

## Supplementary Information

**Additional file 1.** Terms used for database search.

## Data Availability

Original data used to perform the analysis presented here are publicly available on https://fis.fda.gov/extensions/FPD-QDE-FAERS/FPD-QDE-FAERS.html.

## Abbreviations

AF: Atrial fibrillation
ATC: Anatomic therapeutic classification
CI: Confidence interval
CVD: Cardiovascular disease
DKA: Diabetic ketoacidosis
FAERS: Food and Drug Administration adverse event reporting system
FDA: Food and Drug Administration
FG: Fournier’s gangrene
GUTI: Genitourinary tract infections
HF: Heart failure
HFrEF: Heart failure with reduced ejection fraction
PRR: Proportional reporting ratios
RCT: Randomized controlled trials
SGLT2i: Sodium glucose cotransporter-2 inhibitors
T2D: Type 2 diabetes

## References

[1] Zaccardi F, Webb DR, Htike ZZ, Youssef D, Khunti K, Davies MJ (2016). Efficacy and safety of sodium–glucose co-transporter-2 inhibitors in type 2 diabetes mellitus: systematic review and network meta-analysis. Diabetes Obes Metab.

[2] Zelniker TA, Wiviott SD, Raz I, Im K, Goodrich EL, Bonaca MP, Mosenzon O, Kato ET, Cahn A, Furtado RHM (2019). SGLT2 inhibitors for primary and secondary prevention of cardiovascular and renal outcomes in type 2 diabetes: a systematic review and meta-analysis of cardiovascular outcome trials. Lancet.

[3] Packer M, Anker SD, Butler J, Filippatos G, Pocock SJ, Carson P, Januzzi J, Verma S, Tsutsui H, Brueckmann M (2020). Cardiovascular and renal outcomes with empagliflozin in heart failure. N Engl J Med.

[4] McMurray JJV, Solomon SD, Inzucchi SE, Kober L, Kosiborod MN, Martinez FA, Ponikowski P, Sabatine MS, Anand IS, Belohlavek J (2019). Dapagliflozin in patients with heart failure and reduced ejection fraction. N Engl J Med.

[5] Zelniker TA, Bonaca MP, Furtado RHM, Mosenzon O, Kuder JF, Murphy SA, Bhatt DL, Leiter LA, McGuire DK, Wilding JPH (2020). Effect of dapagliflozin on atrial fibrillation in patients with type 2 diabetes mellitus: insights from the DECLARE-TIMI 58 trial. Circulation.

[6] Okunrintemi V, Mishriky BM, Powell JR, Cummings DM (2020). Sodium–glucose co-transporter-2 inhibitors and atrial fibrillation in the cardiovascular and renal outcome trials. Diabetes Obes Metab.

[7] Li WJ, Chen XQ, Xu LL, Li YQ, Luo BH (2020). SGLT2 inhibitors and atrial fibrillation in type 2 diabetes: a systematic review with meta-analysis of 16 randomized controlled trials. Cardiovasc Diabetol.

[8] Nichols GA, Reinier K, Chugh SS (2009). Independent contribution of diabetes to increased prevalence and incidence of atrial fibrillation. Diabetes Care.

[9] Huxley RR, Filion KB, Konety S, Alonso A (2011). Meta-analysis of cohort and case-control studies of type 2 diabetes mellitus and risk of atrial fibrillation. Am J Cardiol.

[10] Berg DD, Wiviott SD, Scirica BM, Gurmu Y, Mosenzon O, Murphy SA, Bhatt DL, Leiter LA, McGuire DK, Wilding JPH (2019). Heart failure risk stratification and efficacy of sodium–glucose cotransporter-2 inhibitors in patients with type 2 diabetes mellitus. Circulation.

[11] Goudis CA, Korantzopoulos P, Ntalas IV, Kallergis EM, Liu T, Ketikoglou DG (2015). Diabetes mellitus and atrial fibrillation: pathophysiological mechanisms and potential upstream therapies. Int J Cardiol.

[12] Birkeland KI, Jorgensen ME, Carstensen B, Persson F, Gulseth HL, Thuresson M, Fenici P, Nathanson D, Nystrom T, Eriksson JW (2017). Cardiovascular mortality and morbidity in patients with type 2 diabetes following initiation of sodium–glucose co-transporter-2 inhibitors versus other glucose-lowering drugs (CVD-REAL Nordic): a multinational observational analysis. Lancet Diabetes Endocrinol.

[13] Persson F, Nystrom T, Jorgensen ME, Carstensen B, Gulseth HL, Thuresson M, Fenici P, Nathanson D, Eriksson JW, Norhammar A (2018). Dapagliflozin is associated with lower risk of cardiovascular events and all-cause mortality in people with type 2 diabetes (CVD-REAL Nordic) when compared with dipeptidyl peptidase-4 inhibitor therapy: a multinational observational study. Diabetes Obes Metab.

[14] Ling AW, Chan CC, Chen SW, Kao YW, Huang CY, Chan YH, Chu PH (2020). The risk of new-onset atrial fibrillation in patients with type 2 diabetes mellitus treated with sodium glucose cotransporter 2 inhibitors versus dipeptidyl peptidase-4 inhibitors. Cardiovasc Diabetol.

[15] Chretien B, Jourdan JP, Davis A, Fedrizzi S, Bureau R, Sassier M, Rochais C, Alexandre J, Lelong-Boulouard V, Dolladille C (2020). Disproportionality analysis in VigiBase(R) as a drug repositioning method for the discovery of potentially useful drugs in Alzheimer’s disease. Br J Clin Pharmacol.

[16] Zaza P, Matthieu R, Jean-Luc C, Charles K (2020). Drug repurposing in Raynaud’s phenomenon through adverse event signature matching in the World Health Organization pharmacovigilance database. Br J Clin Pharmacol.

[17] Khouri C, Petit C, Tod M, Lepelley M, Revol B, Roustit M, Cracowski JL (2021). Adverse drug reaction risks obtained from meta-analyses and pharmacovigilance disproportionality analyses are correlated in most cases. J Clin Epidemiol.

[18] Raschi E, Poluzzi E, Fadini GP, Marchesini G, De Ponti F (2018). Observational research on sodium glucose co-transporter-2 inhibitors: a real breakthrough?. Diabetes Obes Metab.

[19] Fadini GP, Sarangdhar M, De Ponti F, Avogaro A, Raschi E (2019). Pharmacovigilance assessment of the association between Fournier’s gangrene and other severe genital adverse events with SGLT-2 inhibitors. BMJ Open Diabetes Res Care.

[20] Fadini GP, Bonora BM, Avogaro A (2017). SGLT2 inhibitors and diabetic ketoacidosis: data from the FDA adverse event reporting system. Diabetologia.

[21] Fadini GP, Sarangdhar M, Avogaro A (2018). Pharmacovigilance evaluation of the association between DPP-4 inhibitors and heart failure: stimulated reporting and moderation by drug interactions. Diabetes Ther.

[22] Sarangdhar M, Tabar S, Schmidt C, Kushwaha A, Shah K, Dahlquist JE, Jegga AG, Aronow BJ (2016). Data mining differential clinical outcomes associated with drug regimens using adverse event reporting data. Nat Biotechnol.

[23] Raschi E, Poluzzi E, Salvo F, Pariente A, De Ponti F, Marchesini G, Moretti U (2018). Pharmacovigilance of sodium–glucose co-transporter-2 inhibitors: what a clinician should know on disproportionality analysis of spontaneous reporting systems. Nutr Metab Cardiovasc Dis.

[24] Alonso A, Lopez FL, Matsushita K, Loehr LR, Agarwal SK, Chen LY, Soliman EZ, Astor BC, Coresh J (2011). Chronic kidney disease is associated with the incidence of atrial fibrillation: the Atherosclerosis Risk in Communities (ARIC) study. Circulation.

[25] Davies MJ, D’Alessio DA, Fradkin J, Kernan WN, Mathieu C, Mingrone G, Rossing P, Tsapas A, Wexler DJ, Buse JB (2018). Management of hyperglycemia in type 2 diabetes, 2018. A consensus report by the American Diabetes Association (ADA) and the European Association for the Study of Diabetes (EASD). Diabetes Care.

[26] Arnaud M, Salvo F, Ahmed I, Robinson P, Moore N, Begaud B, Tubert-Bitter P, Pariente A (2016). A method for the minimization of competition bias in signal detection from spontaneous reporting databases. Drug Saf.

[27] Xiao L, Salem JE, Clauss S, Hanley A, Bapat A, Hulsmans M, Iwamoto Y, Wojtkiewicz G, Cetinbas M, Schloss MJ (2020). Ibrutinib-mediated atrial fibrillation due to inhibition of CSK. Circulation.

[28] Liu M, McPeek Hinz ER, Matheny ME, Denny JC, Schildcrout JS, Miller RA, Xu H (2013). Comparative analysis of pharmacovigilance methods in the detection of adverse drug reactions using electronic medical records. J Am Med Inform Assoc.

[29] Benjamin EJ, Wolf PA, D’Agostino RB, Silbershatz H, Kannel WB, Levy D (1998). Impact of atrial fibrillation on the risk of death: the Framingham heart study. Circulation.

[30] Ott A, Breteler MM, de Bruyne MC, van Harskamp F, Grobbee DE, Hofman A (1997). Atrial fibrillation and dementia in a population-based study. The Rotterdam study. Stroke.

[31] Wolf PA, Dawber TR, Thomas HE, Kannel WB (1978). Epidemiologic assessment of chronic atrial fibrillation and risk of stroke: the Framingham study. Neurology.

[32] Benjamin EJ, Chen PS, Bild DE, Mascette AM, Albert CM, Alonso A, Calkins H, Connolly SJ, Curtis AB, Darbar D (2009). Prevention of atrial fibrillation: report from a national heart, lung, and blood institute workshop. Circulation.

[33] Heerspink HJL, Stefansson BV, Correa-Rotter R, Chertow GM, Greene T, Hou FF, Mann JFE, McMurray JJV, Lindberg M, Rossing P (2020). Dapagliflozin in patients with chronic kidney disease. N Engl J Med.

[34] Michaud GF, Stevenson WG (2021). Atrial fibrillation. N Engl J Med.

[35] Bonora BM, Avogaro A, Fadini GP (2020). Extraglycemic effects of SGLT2 inhibitors: a review of the evidence. Diabetes Metab Syndr Obes.

[36] Shao Q, Meng L, Lee S, Tse G, Gong M, Zhang Z, Zhao J, Zhao Y, Li G, Liu T (2019). Empagliflozin, a sodium glucose co-transporter-2 inhibitor, alleviates atrial remodeling and improves mitochondrial function in high-fat diet/streptozotocin-induced diabetic rats. Cardiovasc Diabetol.

[37] Peng X, Li L, Zhang M, Zhao Q, Wu K, Bai R, Ruan Y, Liu N (2020). Sodium–glucose cotransporter 2 inhibitors potentially prevent atrial fibrillation by ameliorating ion handling and mitochondrial dysfunction. Front Physiol.

[38] Yurista SR, Sillje HHW, Rienstra M, de Boer RA, Westenbrink BD (2020). Sodium–glucose co-transporter 2 inhibition as a mitochondrial therapy for atrial fibrillation in patients with diabetes?. Cardiovasc Diabetol.

