# Supplementary material for: SGLT-2 inhibitors and atrial fibrillation in the Food and Drug Administration adverse event reporting system
Source: Cardiovasc Diabetol. 2021 Feb 11;20:39. doi: 10.1186/s12933-021-01243-4 (PMC7879696; doi:10.1186/s12933-021-01243-4)
Supplement: Supplementary file 1 — Additional file 1. Terms used for database search. [file 12933_2021_1243_MOESM1_ESM.docx]

**SGLT2i**

"canagliflozin" OR "empagliflozin" OR "dapagliflozin" OR "metformin and canagliflozin" OR "linagliptin and empagliflozin" OR "metformin and empagliflozin" OR "ertugliflozin" OR "ipragliflozin" OR "metformin and dapagliflozin" OR "sotagliflozin"

**ATC10 + insulin**

"sitagliptin" OR "metformin and sitagliptin" OR "linagliptin" OR "saxagliptin" OR "vildagliptin" OR "metformin and vildagliptin" OR "alogliptin" OR "metformin and saxagliptin" OR "metformin and linagliptin" OR "metformin and alogliptin" OR "pioglitazone and alogliptin" OR "evogliptin" OR "gemigliptin" OR "phenformin" OR "metformin" OR "metformin and rosiglitazone" OR "metformin and pioglitazone" OR "metformin and repaglinide" OR "metformin and acarbose" OR "metformin and sulfonylureas" OR "glibenclamide" OR "chlorpropamide" OR "tolbutamide" OR "glibornuride" OR "tolazamide" OR "carbutamide" OR "glipizide" OR "metformin and sulfonylureas" OR "gliquidone" OR "gliclazide" OR "glisoxepide" OR "glimepiride" OR "glimepiride and rosiglitazone" OR "glimepiride and pioglitazone" OR "acetohexamide" OR "glymidine" OR "acarbose" OR "metformin and acarbose" OR "miglitol" OR "voglibose" OR "troglitazone" OR "rosiglitazone" OR "metformin and rosiglitazone" OR "glimepiride and rosiglitazone" OR "pioglitazone" OR "metformin and pioglitazone" OR "glimepiride and pioglitazone" OR "pioglitazone and alogliptin" OR "exenatide" OR "liraglutide" OR "lixisenatide" OR "albiglutide" OR "dulaglutide" OR "semaglutide" OR "guar gum" OR "repaglinide" OR "metformin and repaglinide" OR "nateglinide" OR "pramlintide" OR "benfluorex" OR "mitiglinide" OR "tolrestat" OR "insulins and analogues" OR "insulins and analogues for injection, long-acting" OR "insulins and analogues for injection, fast-acting" OR "insulins and analogues for injection, intermediate- or long-acting combined with fast-acting" OR "insulins and analogues for injection, intermediate-acting" OR "insulin glargine" OR "insulin (human)" OR "insulins and analogues for inhalation" OR "insulin lispro" OR "insulin aspart" OR "insulin detemir" OR "insulin glulisine" OR "insulin (beef)" OR "insulin degludec" OR "insulin (pork)" OR "insulin degludec and insulin aspart" OR "insulin degludec and liraglutide"

**Antiarrhythmics**

"amiodarone" OR "flecainide" OR "propafenone" OR "dronedarone" OR "verapamil" OR "diltiazem" OR "sotalol" OR "carvedilol" OR "metoprolol" OR "atenolol" OR "bisoprolol" OR "nebivolol" OR "propranolol" OR "digoxin" OR "digitoxin"

**Diabetes indication**

"glucose metabolism disorders (incl diabetes mellitus)" OR "diabetes mellitus (incl subtypes)" OR "diabetes mellitus" OR "type 2 diabetes mellitus" OR "diabetes mellitus non-insulin-dependent" OR "type 1 diabetes mellitus" OR "carbohydrate tolerance analyses (incl diabetes)" OR "insulin-requiring type 2 diabetes mellitus" OR "diabetes mellitus insulin-dependent" OR "gestational diabetes" OR "insulin-requiring type ii diabetes mellitus" OR "diabetes mellitus inadequate control" OR "diabetes mellitus management" OR "diabetes" OR "type ii diabetes mellitus" OR "diabetes prophylaxis" OR "latent autoimmune diabetes in adults" OR "insulin resistant diabetes" OR "type i diabetes mellitus" OR "cystic fibrosis related diabetes" OR "type 3 diabetes mellitus" OR "pancreatogenous diabetes" OR "diabetes mellitus malnutrition-related" OR "diabetes mellitus" OR "fulminant type 1 diabetes mellitus" OR "insulin-dependent diabetes mellitus" OR "non-insulin-dependent diabetes mellitus" OR "diabetes complicating pregnancy" OR "type ii diabetes" OR "monogenic diabetes" OR "prediabetes" OR "diabetes mellitus nos" OR "pre-diabetes" OR "acquired lipoatrophic diabetes" OR "borderline diabetes" OR "diabetes mellitus loss of control" OR "diabetes mellitus poor control" OR "diabetes mellitus without mention of complication" OR "diabetes steroid-induced" OR "diabetes with renal manifestations"

**Atrial arrhythmia**

"atrial fibrillation" OR "atrial flutter"

**Stroke**

"ischaemic stroke" OR "haemorrhagic stroke" OR "embolic stroke" "haemorrhagic transformation stroke" OR "thrombotic stroke" OR "brain stem stroke" OR "stroke in evolution" OR "post procedural stroke" OR "lacunar stroke" OR "basal ganglia stroke" OR "cerebellar stroke" OR "thromboembolic stroke" OR "nih stroke scale score increased" OR "nih stroke scale abnormal" OR "stroke volume increased" OR "vertebrobasilar stroke"

**Heart failure**

"cardiac failure" OR "cardiac failure acute" OR "cardiac failure chronic" OR "cardiac failure congestive" OR "cardiac failure high output" OR "cardiogenic shock" OR "low cardiac output syndrome" OR "cardio-respiratory distress" OR "cardiopulmonary failure" OR "cardiac cirrhosis" OR "ventricular failure" OR "cardiorenal syndrome" OR "cardiac asthma" OR "acute pulmonary oedema" OR "left ventricular failure" OR "pulmonary congestion" OR "pulmonary oedema" OR "acute left ventricular failure" OR "chronic left ventricular failure" OR "cor pulmonale" OR "cor pulmonale acute" OR "cor pulmonale chronic" OR "right ventricular failure" OR "acute right ventricular failure" OR "chronic right ventricular failure"

**Genitourinary tract infection**

"urinary tract signs and symptoms" OR "urinary tract infections" OR "genitourinary tract disorders nec" OR "genitourinary tract infections and inflammations nec" OR "urinary tract infection" OR "urinary tract signs and symptoms nec" OR "genital and urinary tract disorders nec" OR "escherichia urinary tract infection" OR "menopausal effects on the genitourinary tract" OR "urinary tract disorder" OR "haemorrhage urinary tract" OR "urinary tract infection bacterial" OR "urinary tract infection enterococcal" OR "urinary tract infection pseudomonal" OR "urinary tract infection staphylococcal" OR "urinary tract infection fungal" OR "urinary tract pain" OR "urinary tract inflammation" OR "lower urinary tract symptoms" OR "genitourinary tract infection" OR "streptococcal urinary tract infection" OR "urinary tract infection viral" OR "urinary tract discomfort" OR "urinary tract injury" OR "genitourinary tract gonococcal infection" OR "urinary tract abscess" OR "cytomegalovirus urinary tract infection" OR "genitourinary tract disorders nec" OR "genitourinary tract infections and inflammations nec" OR "menopausal effects on the genitourinary tract" OR "genitourinary tract infection" OR "tuberculosis of genitourinary system" OR "genitourinary symptom" OR "genitourinary chlamydia infection" OR "genitourinary tract gonococcal infection" OR "somatoform genitourinary disorder"

**Fournier’s gangrene**

"fournier's gangrene" OR "scrotal gangrene" OR "scrotal abscess" OR "perineal abscess" OR "perineal cellulitis" OR "perineal infection" OR "necrotising fasciitis" OR "necrotising fasciitis streptococcal" OR "necrotising fasciitis staphylococcal" OR "gangrenous balanitis" OR "vulval cellulitis" OR "cellulitis of male external genital organ"

**Amputations**

"amputation" OR "limb amputation" OR "leg amputation" OR "foot amputation" OR "finger amputation" OR "toe amputation" OR "arm amputation" OR "hand amputation"

**Cardiac disorders indication**

"heart failures" OR "heart failures nec" OR "heart rate and pulse investigations" OR "heart failure signs and symptoms" OR "heart transplant" OR "ischaemic heart disease prophylaxis" OR "heart rate irregular" OR "heart rate increased" OR "heart disease congenital" OR "heart valve replacement" OR "heart rate" OR "heart rate abnormal" OR "hypertensive heart disease" OR "heart rate decreased" OR "heart valve incompetence" OR "heart transplant rejection" OR "heart valve operation" OR "heart and lung transplant" OR "heart disorder" OR "rheumatic heart disease" OR "congestive heart failure" OR "hypoplastic left heart syndrome" OR "heart block congenital" OR "nuclear magnetic resonance imaging heart" OR "chronic pulmonary heart disease" OR "heartburn" OR "heart injury" OR "univentricular heart" OR "heart attack" OR "heart failure" OR "foetal heart rate decreased" OR "heart alternation" OR "artificial heart implant" OR "coronary heart disease" OR "metastases to heart" OR "complications of transplanted heart" OR "congenital heart valve disorder" OR "heart valve stenosis" OR "hyperkinetic heart syndrome" OR "hypoplastic right heart syndrome" OR "ischaemic heart disease" OR "ischemic heart disease" OR "artificial heart device user" OR "heartbeats irregular" OR "open heart surgery" OR "heart disease, unspecified" OR "heart rate normal" OR "heart sounds abnormal" OR "heart valve insufficiency" OR "malignant hypertensive heart disease" OR "carcinoid heart disease" OR "chronic ischemic heart disease, unspecified" OR "foetal heart rate abnormal" OR "heart valve calcification" OR "heartbeats increased" OR "syphilitic endocarditis of heart valve" OR "chronic heart failure" OR "foetal heart rate increased" OR "heart valve disorders" OR "right heart failure" OR "biopsy heart" OR "chronic ischaemic heart disease, unspecified" OR "chronic pulmonary heart disease, unspecified" OR "congenital heart disease nos" OR "foetal heart rate disorder" OR "heart fluttering" OR "heart rate high" OR "heart sounds" OR "heart valve explantation" OR "heart valve replacement nos" OR "irregular heartbeat" OR "ischemic heart disease prophylaxis" OR "kyphoscoliotic heart disease" OR "prophylaxis against heart transplant rejection" OR "regulate heart rate" OR "valvular heart disease nos" OR "cardiac disorders" OR "cardiac arrhythmias" OR "cardiac and vascular investigations (excl enzyme tests)" OR "cardiac disorder signs and symptoms" OR "cardiac disorders nec" OR "cardiac disorder" OR "cardiac failure" OR "cardiac therapeutic procedures" OR "cardiac failure congestive" OR "cardiac signs and symptoms nec" OR "cardiac therapeutic procedures nec" OR "cardiac valve therapeutic procedures" OR "cardiac and vascular disorders congenital" OR "cardiac imaging procedures" OR "ventricular arrhythmias and cardiac arrest" OR "cardiac function diagnostic procedures" OR "cardiac failure chronic" OR "cardiac valve disorders" OR "cardiac operation" OR "congenital cardiac disorders" OR "cardiac disorders congenital nec" OR "cardiac infections" OR "congenital cardiac structural defects nec" OR "cardiac and vascular procedural complications" OR "catheterisation cardiac" OR "cardiac stress test" OR "cardiac valve disorders nec" OR "cardiac septal defects congenital" OR "cardiac valve disease" OR "cardiac device therapeutic procedures" OR "cardiac conduction disorders" OR "cardiac hypertensive complications" OR "cardiac pacemaker insertion" OR "cardiac failure acute" OR "cardiac fibrillation" OR "oedema due to cardiac disease" OR "cardiac arrest" OR "cardiac valve prosthesis user" OR "intracardiac thrombus" OR "cardiac pharmacologic stress test" OR "cardiac auscultatory investigations" OR "cardiac murmur" OR "right-to-left cardiac shunt" OR "cardiac ablation" OR "cardiac assistance device user" OR "cardiac flutter" OR "skeletal and cardiac muscle analyses" OR "cardiac amyloidosis" OR "non-cardiac chest pain" OR "cardiac imaging procedure" OR "congenital cardiac malpositions and transpositions" OR "cardiac valve disorders congenital" OR "multiple cardiac abnormalities congenital" OR "cardiac neoplasms" OR "cardiac neoplasms nec" OR "cardiac ventricular thrombosis" OR "cardiac hypoplasias congenital" OR "cardiac aneurysm" OR "cardiac septal defect" OR "cardiac infections and inflammations nec" OR "cardiac hypertrophy" OR "cardiac discomfort" OR "cardiac sarcoidosis" OR "cardiac infection" OR "foetal cardiac disorder" OR "valvuloplasty cardiac" OR "cardiac valve replacement complication" OR "positive cardiac inotropic effect" OR "cardiac ventricular disorder" OR "cardiac enzymes increased" OR "cardiac neoplasm malignant" OR "cardiac pacemaker replacement" OR "cardiac asthma" OR "cardiac function test" OR "cardiac stress test abnormal" OR "cardiac rehabilitation therapy" OR "cardiac ventriculogram" OR "cardiac output decreased" OR "cardiac tamponade" OR "cardiac ventriculogram left" OR "cardiac aneurysm repair" OR "cardiac output" OR "cardiac disorder prophylaxis" OR "cardiac insufficiency" OR "cardiac neoplasm unspecified" OR "cardiac arrhythmia" OR "cardiac disorder nos" OR "low cardiac output syndrome" OR "cardiac procedure complication" OR "cardiac electrophysiologic study" OR "cardiac myxoma" OR "cardiac pacemaker removal" OR "cardiac function disturbance postoperative" OR "cardiac resynchronisation therapy" OR "cardiac cirrhosis" OR "cardiac complications associated with device" OR "cardiac failure high output" OR "cardiac output increased" OR "cardiac valve vegetation" OR "cardiac pacemaker revision" OR "cardiac dysrhythmias" OR "cardiac enzymes" OR "cardiac function test abnormal" OR "neonatal cardiac failure" OR "benign cardiac neoplasm" OR "cardiac autonomic neuropathy" OR "cardiac catheterisation" OR "cardiac index" OR "cardiac pacemaker battery replacement" OR "cardiac perforation" OR "cardiac pseudoaneurysm" OR "cardiac siderosis" OR "cardiac valve sclerosis" OR "cardiac catheterization" OR "cardiac monitoring abnormal" OR "cardiac murmur functional" OR "insufficiency cardiac" OR "intracardiac mass" OR "primary cardiac lymphoma" OR "accessory cardiac pathway" OR "cardiac malpositions congenital" OR "cardiac monitoring" OR "cardiac septal defect repair" OR "cardiac septal hypertrophy" OR "cardiac transplant" OR "cardiac valve fibroelastoma" OR "catheterisation cardiac abnormal" OR "decompensation cardiac" OR "multiple cardiac defects" OR "myocardiac abscess" OR "post cardiac arrest syndrome" OR "ventriculo-cardiac shunt" OR "acquired cardiac septal defect" OR "cardiac arrhythmia nos" OR "cardiac cath" OR "cardiac dysrhythmia, unspecified" OR "cardiac failure nos" OR "cardiac granuloma" OR "cardiac histopathology procedures" OR "cardiac imaging procedure abnormal" OR "cardiac neurosis" OR "cardiac pacemaker evaluation" OR "cardiac pacemaker malfunction" OR "cardiac septal defect residual shunt" OR "cardiac valve rupture" OR "cardiac valvulopathy" OR "congestive cardiac failure" OR "gastrocardiac syndrome" OR "negative cardiac inotropic effect" OR "pacemaker insertion (cardiac)" OR "sudden cardiac death"

**Renal disease indications**

"renal and urinary disorders" OR "renal failure and impairment" OR "renal failure complications" OR "renal failure chronic" OR "end stage renal disease" OR "renal failure" OR "renal disorder" OR "diabetic complications renal" OR "renal impairment" OR "renal failure acute" OR "diabetic end stage renal disease" OR "renal replacement therapy" OR "renal injury" OR "end stage renal disease (esrd)" OR "renal tubular necrosis" OR "cardiorenal syndrome" OR "renal function test abnormal" OR "acute prerenal failure" OR "creatinine renal clearance decreased" OR "prerenal failure" OR "renal insufficiency" OR "postrenal failure" OR "chronic renal failure anemia" OR "creatinine renal clearance abnormal" OR "chronic renal impairment" OR "chronic renal insufficiency" OR "diabetes with renal manifestations" OR "end stage renal failure" OR "renal disease" OR "renal tubular injury" OR "chronic kidney disease" OR "acute kidney injury" OR "chronic kidney disease-mineral and bone disorder" OR "kidney disorder" OR "kidney fibrosis" OR "kidney failure"
